# Brain-Death in Rats Increases Neutrophil Extracellular Trap Formation in Donor Organs

**DOI:** 10.3389/ti.2025.14223

**Published:** 2025-04-22

**Authors:** Maryna Van Zyl, Roberto Armstrong Junior, Petra Ottens, Harry Van Goor, Mia-Jeanne Van Rooy, Ton Lisman, Henri G. D. Leuvenink, Jan-Luuk Hillebrands

**Affiliations:** ^1^ Pathology Division, Department of Pathology and Medical Biology, University Medical Center Groningen, University of Groningen, Groningen, Netherlands; ^2^ Department of Physiology, School of Medicine, Faculty of Health Sciences, University of Pretoria, Pretoria, South Africa; ^3^ Surgical Research Laboratory, Department of Surgery, University Medical Center Groningen, University of Groningen, Groningen, Netherlands

**Keywords:** donor, brain-death, neutrophils, neutrophil extracellular traps, endothelial activation

## Abstract

During brain-death, increased numbers of neutrophils are recruited to organs as part of the inflammatory response. In the organ microenvironment, the recruited neutrophils may release neutrophil extracellular traps (NETs) through interaction with various pro-inflammatory stimuli, contributing to brain-death-induced endothelial activation, microthrombus formation and ultimately a decline in organ quality. To investigate whether NETs form in organs from brain-dead donors; kidneys, hearts, livers, and plasma samples were collected from brain-dead or sham-operated rats. The presence of NET-specific components, neutrophils and macrophages were analyzed through immunofluorescent microscopy. Endothelial activation and platelet infiltration were analyzed through immunohistochemistry and qRT-PCR analysis. Plasma free thiol levels were used to evaluate systemic oxidative stress. Increased neutrophils, NETs and NET/neutrophil ratios were observed in kidneys, hearts and livers of brain-dead rats compared to sham-operated rats. Numbers of NETs positively correlated with the extent of endothelial cell activation. Brain-dead animals also had increased kidney and liver macrophages, increased infiltrated platelets in the liver, and elevated systemic oxidative stress, compared to sham-operated animals. Our findings established the presence of NETs in organs from a brain-dead donor model and suggest that NETs, alongside increased inflammation and a redox imbalance, might prime organs for microvascular endothelial dysfunction and increased injury during brain-death.

## Graphical Abstract

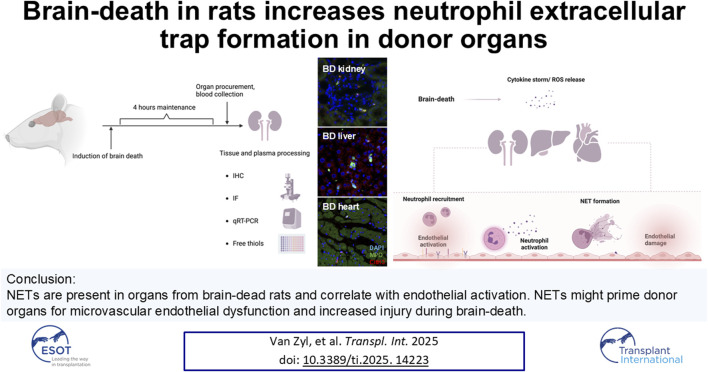

## Introduction

Organ transplantation remains the best treatment option for patients facing end-stage organ failure [1]. Globally, the rise in high disease burden results in an increase in the demand for donor organs, contributing to long waiting lists at transplantation centers [2]. A significant proportion of transplants are performed using grafts from brain-dead (BD) donors [3], however, the quality of these organs may be compromised due to various systemic changes occurring during brain-death [4]. Hemodynamic instability and a build-up in intracranial pressure results in a reduced cardiac output which is overcompensated for by a catecholamine storm, ultimately resulting in the release of a cascade of pro-inflammatory cytokines, organ hypoperfusion [4], activation of complement [5] and coagulation [6], the release of reactive oxygen species (ROS) [7], and increased organ leukocyte infiltration [8]. Increased immune activation thus already starts in the BD donor, which exacerbates ischemia/reperfusion injury (I/R) [9] and potentially also leads to inferior transplant outcome in the recipient compared to living donor transplantation [10, 11]. Finding strategies to modulate immune cell infiltration and activation prior to transplantation might therefore not only enlarge the pool of transplantable organs, but also improve graft survival and transplant outcome.

The pro-inflammatory microenvironment during BD might stimulate neutrophil extracellular trap (NET) formation both within the graft and in the systemic circulation [12, 13]. NETs are characterized by the release of the neutrophil’s nuclear contents to the extracellular space in web-like structures [14]. NET formation may be initiated in response to pathogens [15] or, as is relevant in the context of BD, during sterile inflammation, through stimulation with danger associated molecular patterns (DAMPs) [16], pro-inflammatory cytokines [17], and activated platelets [18]. Following neutrophil stimulation, histones undergo decondensation, mediated by the activity of cytoplasmic enzymes, myeloperoxidase (MPO) or neutrophil elastase (NE), or citrullination through peptidyl arginine deiminase 4 (PAD4) [19]. NET formation culminates in nuclear disintegration or blebbing, and the release of webbed structures containing histones, DNA, MPO and NE [15]. The components of NETs are cytotoxic and often cause extensive tissue damage [16, 20], endothelial cell activation, death [21, 22] and increased vascular permeability [23], the activation of coagulation and complement, and the recruitment of additional immune cells [24]. A mounting body of evidence suggests a role of NET formation in transplantation-related complications in various organs [25, 26]. NETs have been associated with acute antibody-mediated rejection in transplanted kidneys [25], primary graft dysfunction in lungs [27], and acute liver rejection [28]. However, the presence of NETs in organs from BD donors remains unexplored.

By using a rat BD model, we aimed to explore NET formation in organs during BD, hypothesizing that NET formation already starts in the donor. This might cause damage prior to transplantation and potentially exacerbate I/R injury following implantation, contributing to delayed graft function and graft failure since NETs have been shown to be associated with acute kidney injury [16].

Furthermore, it has been established that the endothelium undergoes various changes during brain-death, including the increased expression of adhesion molecules involved in neutrophil recruitment, and transformation to a pro-inflammatory, pro-coagulant state resulting in increased platelet activation and adhesion [6, 7]. Brain-death is also accompanied by progressive systemic oxidative stress through gradual, cumulative release of ROS by stressed, hypoxic cells [7, 29].

During brain-death, endothelial activation, already observed early during the onset of BD [7, 30], might increase neutrophil recruitment and consequently also increase NET formation, enhancing organ injury through endothelial damage [31] and thrombus formation [32]. Neutrophils might not only be primed for increased NET formation through ROS release by other cells, but NETs might also contribute to the gradual onset of the systemic oxidative burden during brain-death [33].

We therefore also aimed to investigate the association between NET formation and endothelial activation, platelet infiltration, and ROS production during brain-death in our model.

## Materials and Methods

### Animal Maintenance

In this post-hoc study, eight-week-old, male and female Wistar F344/IcoCrl rats (Charles River, Italy) were used for all experiments. Prior to experiments, the rats were allowed free access to water and food and maintained at ambient room temperature with a 12 h light/dark cycle. During care of the animals, *The Principles of Laboratory Animal Care* were followed. The local animal ethics committee approved the protocol in accordance with the Experiments on Animals Act and ARRIVE guidelines (IvD 171245-01-002).

### Brain-Death (BD) Rat Model and Sample Collection

Rats in the BD group (n = 18, 10 males and 8 females) were subjected to slow-induced BD by simulating cerebral hemorrhage through intracranial balloon catheterization and inflation, which was maintained for 4 h as previously described [34], visually depicted in Supplementary Figure S1 and detailed in Supplementary Digital Content. Sham-operated animals (n = 16, 8 males and 8 females) received the same cranial perforation but without BD induction. Organs were either flash frozen, or formalin-fixed and paraffin-embedded (FFPE) for analysis. Plasma samples were obtained by centrifuging heparinized whole blood, collected from the abdominal aorta, at 1,200 x *g* for 10 min at 4°C. Frozen tissue or plasma samples were stored at −80°C until use.

### Immunofluorescence

The co-localization of DNA, MPO and citrullinated histone 3 (CitH3) was used to identify NETs, while an antibody against CD68 was used to identify monocytes and macrophages to correct for MPO expressing macrophages. FFPE rat kidneys, hearts and livers were sectioned into 3 µm sections and mounted onto slides. The slides were deparaffinized in xylene, rehydrated in gradient steps of alcohol, submitted to 180 min of heat-induced antigen retrieval at 60°C in a citrate buffer (pH 6.0) and blocked for 1 h with 5% bovine serum albumin (BSA). The tissue was incubated with primary antibodies against CitH3 (1:100, ab5103, Abcam, UK), MPO (1:200, AF3667, Novus Biologicals, United States) and CD68, clone ED-1 (1:100, MCA341GA, BIO-RAD) for 90 min at room temperature followed by incubation with donkey anti-rabbit Alexa-Fluor^®^ 647 (1:500), donkey anti-goat Alexa-Fluor^®^ 488 (1:500), and donkey anti-mouse Alexa-Fluor^®^ 586 (1:500) from Abcam (UK). The slides were mounted with Vectashield antifade mounting medium (Vector Laboratories, United States) containing DAPI (ThermoFisher Scientific, United States) and digitized with an Olympus VS200 Fluorescent Slide Scanner (Olympus, Japan). NETs were quantified with Qupath (v. 0.4.1.) and expressed as the number of MPO^+^CitH3^+^ double positive but CD68 negative cells per area, or the number of CD68^+^ cells (macrophages) per area. To compare different organs, NETs were expressed as fold increase from sham. Technical details on reagents, antibodies and methods used during immunofluorescence can be found in the Supplementary Digital Content, Supplementary Tables S1, S2.

### Immunohistochemistry

Endothelial activation in kidney, heart and liver sections was analyzed by quantifying protein expression of intercellular adhesion molecule 1 (ICAM-1) and vascular cell adhesion molecule 1 (VCAM-1). Frozen kidney, liver and heart sections were cut in 3 µm sections, dried and fixed with acetone. The sections were blocked with 3% H_2_O_2_ and 5% BSA followed by incubation with mouse anti-ICAM-1 (1:100) from BD Pharmingen and mouse anti-VCAM1 (1:100) from Biogen. The primary antibodies were detected through horseradish peroxidase (HRP)-conjugated secondary and tertiary antibodies [goat anti-mouse-HRP and rabbit anti-goat-HRP [(both 1:100) from DAKO, Denmark] and visualized with 3,3′-Diaminobenzidine (DAB). The sections were counterstained with hematoxylin and dehydrated before mounting with Dibutylphthalate Polystyrene Xylene mounting medium.

For quantification of platelets, rat kidney, liver and heart FFPE sections were deparaffined, rehydrated, submitted to 15 min of heat-induced antigen retrieval at 95°C in a citrate buffer (pH 6) and blocked as described for ICAM-1 and VCAM-1. The tissue was incubated with a rabbit CD41 antibody (1:500, Proteintech, United States) for 1 h. Primary antibody binding was detected with goat anti-rabbit-HRP (1:100) and with rabbit anti-goat-HRP secondary antibodies from DAKO (Denmark) followed by incubation with DAB, a counterstain with hematoxylin and mounting. All tissues were digitized with a Hamamatsu NanoZoomer Digital slide scanner (Hamamatsu, Japan). The expression of ICAM-1, VCAM-1, and platelets were analyzed as the percentage positive area with Fiji/ImageJ (v. 3). Antibody and reagent details are specified in Supplementary Digital Content, Supplementary Tables S1, S3.

### mRNA Analyses

The mRNA expression levels of endothelial adhesion molecules (ICAM-1, VCAM-1, and E-selectin), were analyzed through SYBR™ Green qRT-PCRs. Intron-spanning primers for the target genes were designed through Primer-Blast (NIH, United States) and checked for potential hairpins or dimers with an Oligonucleotide properties calculator. Forward and reverse primers used for genes of interest are summarized in Table 1. RNA was isolated from frozen sections of rat kidneys, hearts and livers using TRIzol^®^ and stored at −20°C until use. The purity, quantity and quality of the isolated RNA were evaluated with a NanoDrop spectrophotometer and agarose gel electrophoresis. Complementary DNA (cDNA) was synthesized using Random primer hexamers and Superscript II (ThermoFisher Scientific, United States). The samples were loaded onto PCR microplates with a master mix containing SYBR™ Green (ThermoFisher Scientific) and gene-specific primers. Amplification of the targets was done with a Roche LightCycler^®^ 480 System (Roche, Switzerland). Melt-curves and Ct values were obtained through Roche LightCycler 480 software (v 1.2.9.11). The relative expression of target genes was normalized against ß-actin expression. Technical details of qRT-PCRs are summarized in Supplementary Digital Content, Supplementary Table S4.

**TABLE 1:** Primer sequences for qRT-PCR.

Gene	Sequence
*Β-actin*	5′-GGAAATCGTGCGTGACATTAAA-3′ 5′- GCGGCAGTGGCCATCTC -3′
*ICAM1*	5′-CCTGGAGATGGAGAAGACCTA -3′ 5′-GGGAAGTACCCTGTGAGGTG -3′
*VCAM1*	5′-GCTCCTCTCGGGAAATGCCA -3′ 5′-ACAACGGAATCCCCAACCTGT -3′
*E-selectin*	5′-ACTTGTGAAGCCCCAGCCAA -3′ 5′-TGGCAGCTACTAGCAGGAACG -3′

Abbreviations: ICAM-1, intercellular adhesion molecule 1; VCAM-1, vascular cell adhesion molecule 1.

### Plasma Free Thiol Levels

Free thiol levels were determined in rat plasma to measure systemic oxidative stress as previously described [35]. Rat plasma samples were diluted 20-fold with a 0.1 M Tris buffer (pH 8.2). The standard curve consisted of increasing concentrations of L-Cysteine in a 0.1 M Tris, 10 mM EDTA buffer (pH 8.2). A color reaction in the sample and standard wells was developed through the addition of 1.9 mM DTNB in 0.1 M phosphate buffer (pH 7.0) for 20 min at room temperature. The absorbance was measured at 412 nm with a Clariostar plus microplate reader (BMG Labtech, Germany). Technical details of the assay are specified in Supplementary Digital Content, Supplementary Table S5.

### Statistics

Sample datasets were examined for normality using the Shapiro-Wilk normality test. Subsequently, Welch t-tests were conducted, allowing for unequal standard deviations, for populations exhibiting normal distribution, whereas Mann-Whitney U tests were employed for data displaying non-normal distribution. For analyses involving comparison of more than one group, an ANOVA with Tukey post-hoc analyses and Bonferroni correction, or Kruskal Wallis with Duns post-hoc test and Bonferroni correction was used. Associations between variables were evaluated using either Pearson or Spearman correlation tests based on normality. Results are presented as either mean and standard error of the mean (SEM) or median and interquartile range (IQR). A significance threshold of p < 0.05 was applied for all analyses. Statistical analyses and data visualization were performed using GraphPad Prism (v. 10.0.0).

## Results

### Increased CitH3-Positive Neutrophils in Brain-Dead Kidneys

**FIGURE 1:**
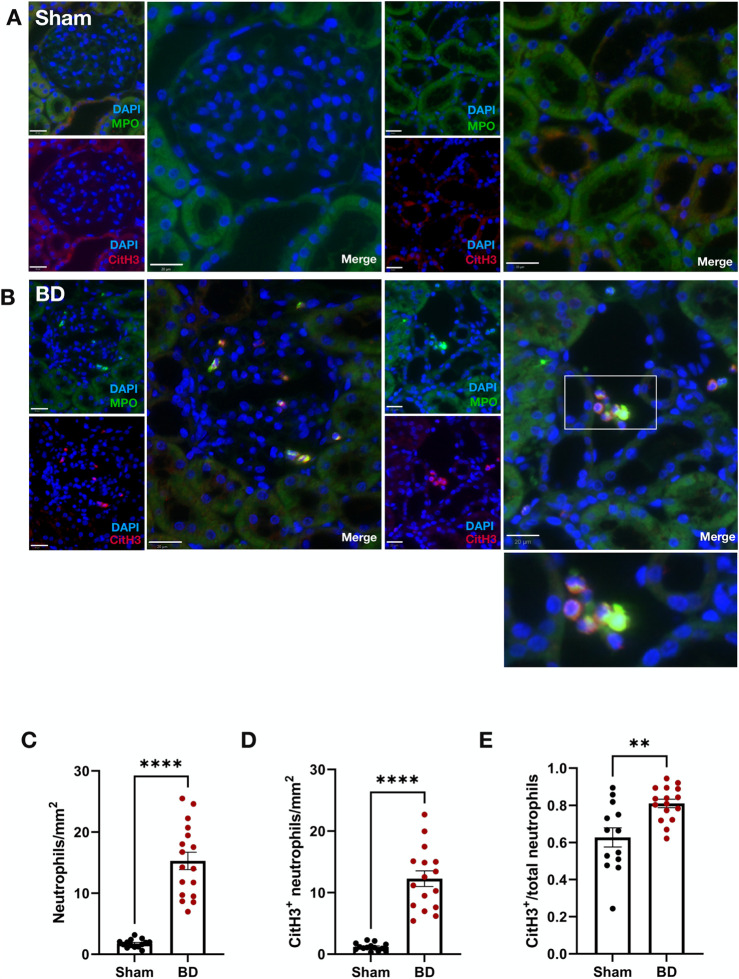
Neutrophil infiltration and NET formation in brain-dead rat kidneys. NET formation was observed in the renal cortex of brain-dead rat kidneys **(B)**, while very few/almost no NETs were observed in kidneys from sham operated animals **(A)**. The brain-dead group had significantly more neutrophil infiltration **(C)**, CitH3 positive neutrophils **(D)**, and CitH3 positive/total neutrophil ratios **(E)** compared to sham. Scale bar equals 20 µm. ****p < 0.0001, **p < 0.01. Data expressed as means (±SEM). Abbreviations: BD, brain-dead; CitH3, citrullinated histone 3; MPO, myeloperoxidase.

Compared to sham-operated animals, (Figure 1A), BD rat kidneys (Figure 1B) had increased neutrophils (MPO^+^) (Figure 1C, p < 0.0001). In BD, significantly more neutrophils were CitH3 positive compared to sham-operated animals (Figure 1D, p < 0.0001), indicative of NET formation. BD rat kidneys also had an increased proportion of CitH3 positive neutrophils (NETs) compared to sham (Figure 1E, p < 0.01). In the BD animals, most NETs were observed in the renal cortex (Figure 1B), located predominantly in the glomeruli, but also in the peritubular capillaries/interstitial spaces.

### Endothelial Activation in Brain-Dead Rat Kidneys Correlates With NET Formation

**FIGURE 2:**
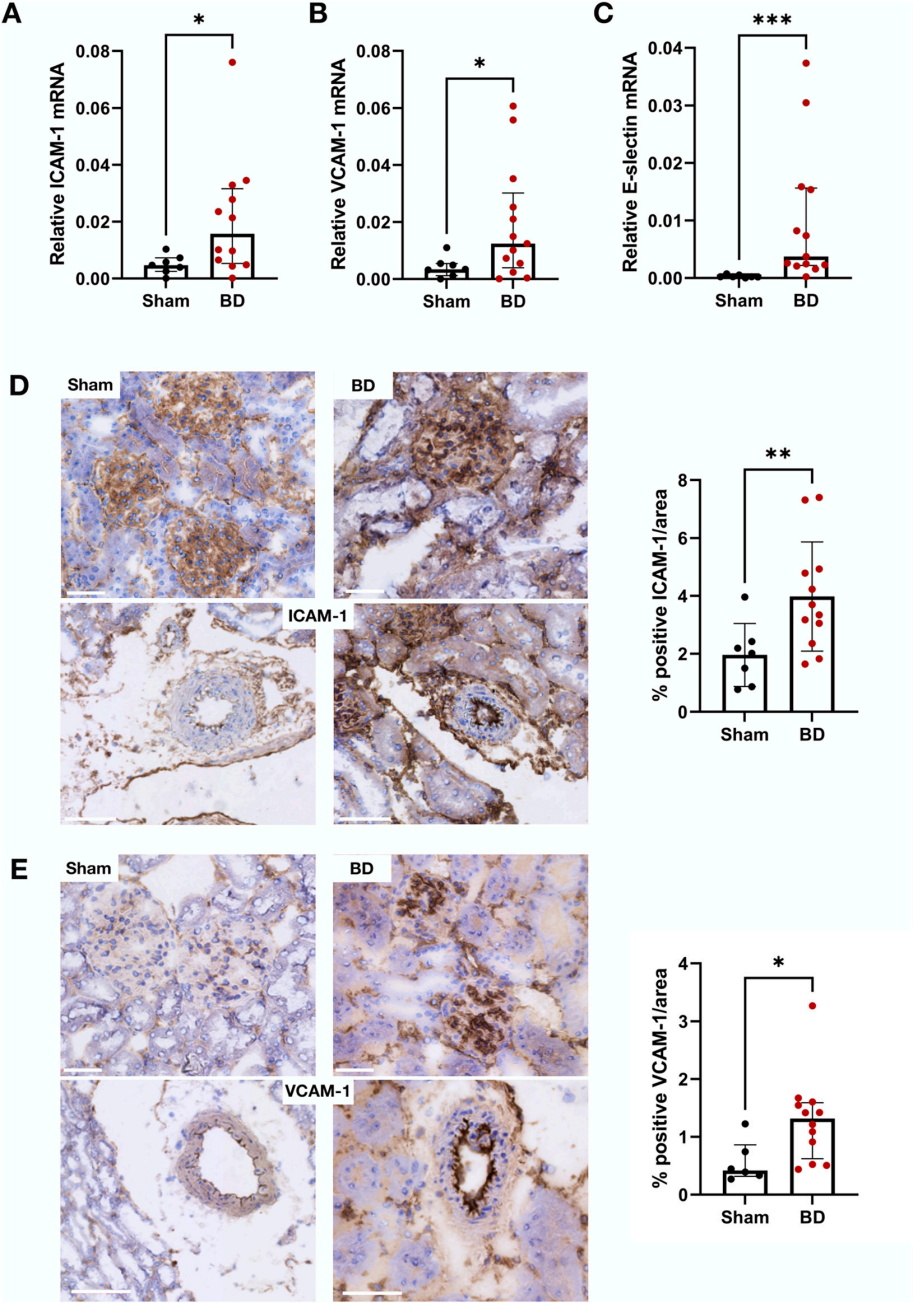
Endothelial activation in brain-dead rat kidneys. Brain-dead rat kidneys had increased ICAM-1 mRNA expression **(A)**, VCAM-1 mRNA expression **(B)** and E-selectin mRNA expression **(C)**. ICAM-1 **(D)** and VCAM-1 **(E)** protein was observed in the glomeruli and vessels and were increased in brain dead animals compared to sham. Scale bar equals 50 µm ***p < 0.001, **p < 0.01, *p < 0.05. Data expressed as median (IQR). Abbreviations: BD, brain-dead, ICAM-1 intercellular cell adhesion molecule 1, VCAM-1, vascular cell adhesion molecule 1.

**FIGURE 3:**
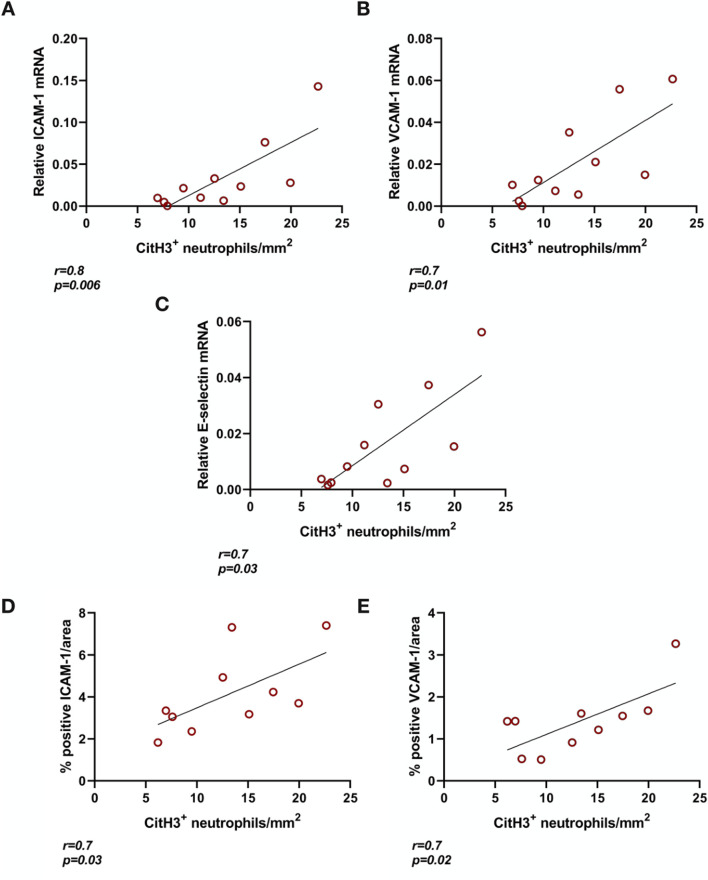
Endothelial activation correlates with NET formation in brain-dead rat kidneys. ICAM-1 **(A)**, VCAM-1 **(B)**, and E-selectin **(C)** mRNA expression as well as ICAM-1 **(D)** and VCAM-1 **(E)** protein in brain-dead rat kidneys positively correlated to NET formation (CitH3 positive neutrophils). Abbreviations: CitH3, citrullinated histone 3; ICAM-1, intercellular cell adhesion molecule 1, MPO, myeloperoxidase, VCAM-1, vascular cell adhesion molecule 1.

**FIGURE 4:**
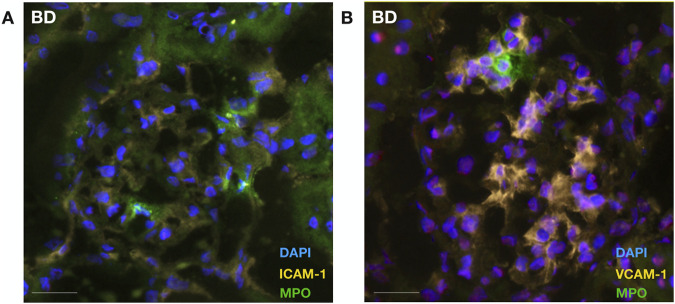
Neutrophil colocalizes with endothelial cellular adhesion molecules in brain-dead rat kidneys. Neutrophil infiltration (MPO) was observed in proximity to ICAM-1 **(A)** and VCAM-1 **(B)** protein expression in the glomeruli of brain-dead rat kidneys. Abbreviations: ICAM-1, intercellular cell adhesion molecule 1; MPO, myeloperoxidase, VCAM-1, vascular cell adhesion molecule 1.

BD rat kidneys had increased endothelial activation associated gene (Figures 2A–C) and protein expression (Figures 2D, E) compared to sham-operated animals. ICAM-1 (Figure 2A), VCAM-1 (Figure 2B) and E-selectin (Figure 2C) mRNA expression levels were elevated compared to sham-operated animals. This was also reflected on the protein level, with increased ICAM-1 (Figure 2D) and VCAM-1 (Figure 2E) protein expression levels in the glomerular capillaries, peritubular capillaries, veins, and arteries of BD rat kidneys. ICAM-1 (Figure 3A), VCAM-1 (Figure 3B) and E-selectin (Figure 3C) mRNA expression, and ICAM-1 (Figure 3D) and VCAM-1 (Figure 3E) protein expression significantly correlated with NET formation in BD rat kidneys. MPO positivity was observed near ICAM-1 (Figure 4A) and VCAM-1 (Figure 4B) protein expression in the glomeruli of BD kidneys.

### Increased CitH3-Positive Neutrophils in Brain-Dead Rat Hearts

**FIGURE 5:**
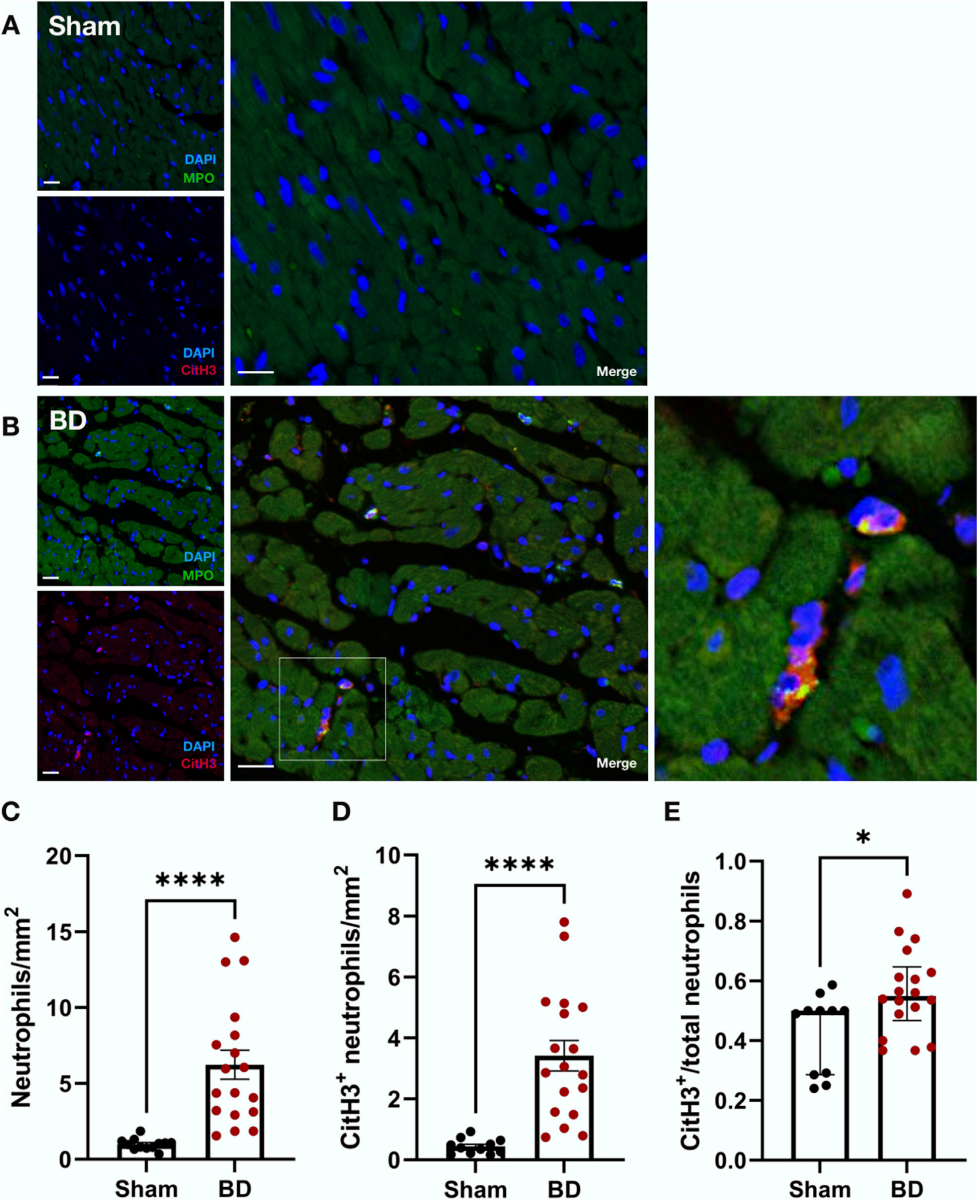
Neutrophil infiltration and NET formation in brain-dead rat hearts. NET formation was observed between myocytes of brain-dead rat hearts **(B)** with very few to almost no neutrophil or NET formation in the hearts of sham operated animals **(A)**. Hearts from brain-dead rats had significantly increased neutrophils **(C)**, CitH3 positive neutrophils **(D)**, and CitH3 positive/total neutrophil ratios **(E)** compared to sham. Scale bar equals 50 µm. ****p < 0.0001,*p < 0.05. Data expressed as means (±SEM), C, D; or median (IQR), E. Abbreviations: BD, brain-death; CitH3, citrullinated histone 3; MPO, myeloperoxidase.

Similar to observations in BD rat kidneys, an increased number of neutrophils (Figure 5C, p < 0.0001) and increased NET formation (CitH3 positive neutrophils, Figure 5D, p < 0.0001) were also observed in BD rat hearts (Figure 5B), compared to hearts from sham-operated animals (Figure 5A). BD rat hearts also had increased NET/neutrophil ratios (Figure 5E, p < 0.05) compared to sham. NET forming neutrophils were observed between myocytes of the myocardium.

### Endothelial Activation Protein in Brain-Dead Hearts Correlates With NET Formation

**FIGURE 6:**
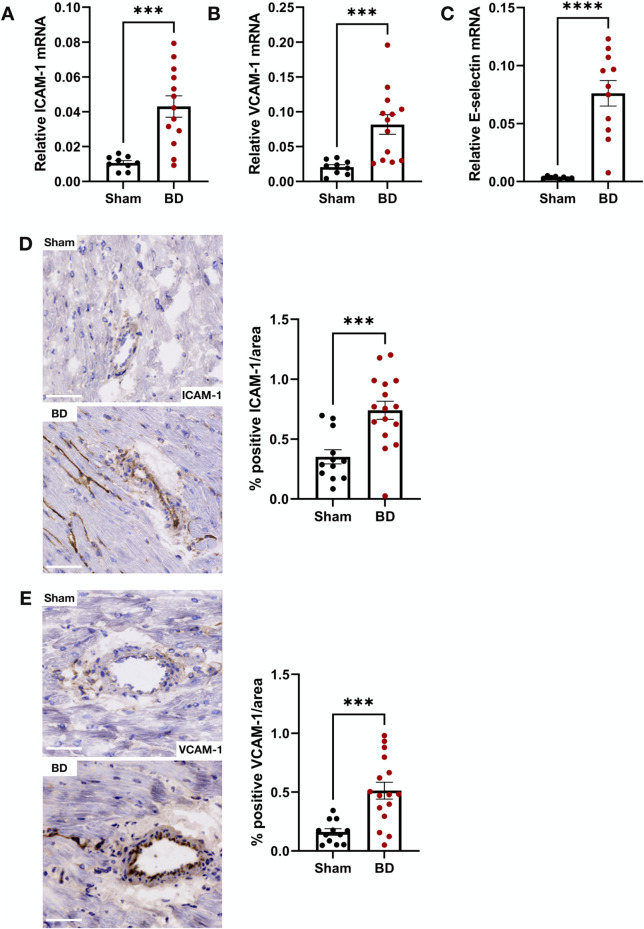
Endothelial activation in brain-dead rat hearts. Brain-dead rats had increased ICAM-1 mRNA **(A)**, VCAM-1 mRNA **(B)** and E-selectin mRNA **(C)** as well as increased ICAM-1 **(D)** and VCAM-1 **(E)** protein levels. ICAM-1 and VCAM-1 was observed both the capillaries and larger vessels **(D, E)**. Scale bar equals 50 µm ****p < 0.0001, ***p < 0.001. Data expressed as means (±SEM). Abbreviations: BD, brain-death; ICAM-1, intercellular cell adhesion molecule 1; VCAM-1, vascular cell adhesion molecule 1.

ICAM-1 (Figure 6A), VCAM-1 (Figure 6B) and E-selectin (Figure 6C) mRNA expression in BD hearts were increased compared to hearts from sham-operated animals. In line with this, BD rat hearts also had increased ICAM-1 (Figure 6D) and VCAM-1 (Figure 6E) protein expression compared to sham. In the BD hearts, both ICAM-1 and VCAM-1 were observed in capillaries and arteries (Figures 6D, E). Protein levels of ICAM-1, positively correlated to NET formation in the BD group (r = 0.7; p = 0.002). Significant correlation between VCAM-1 protein/endothelial activation mRNA and NET formation were not observed (not shown).

### Increased CitH3-Positive Neutrophils in Brain-Dead Rat Livers

**FIGURE 7:**
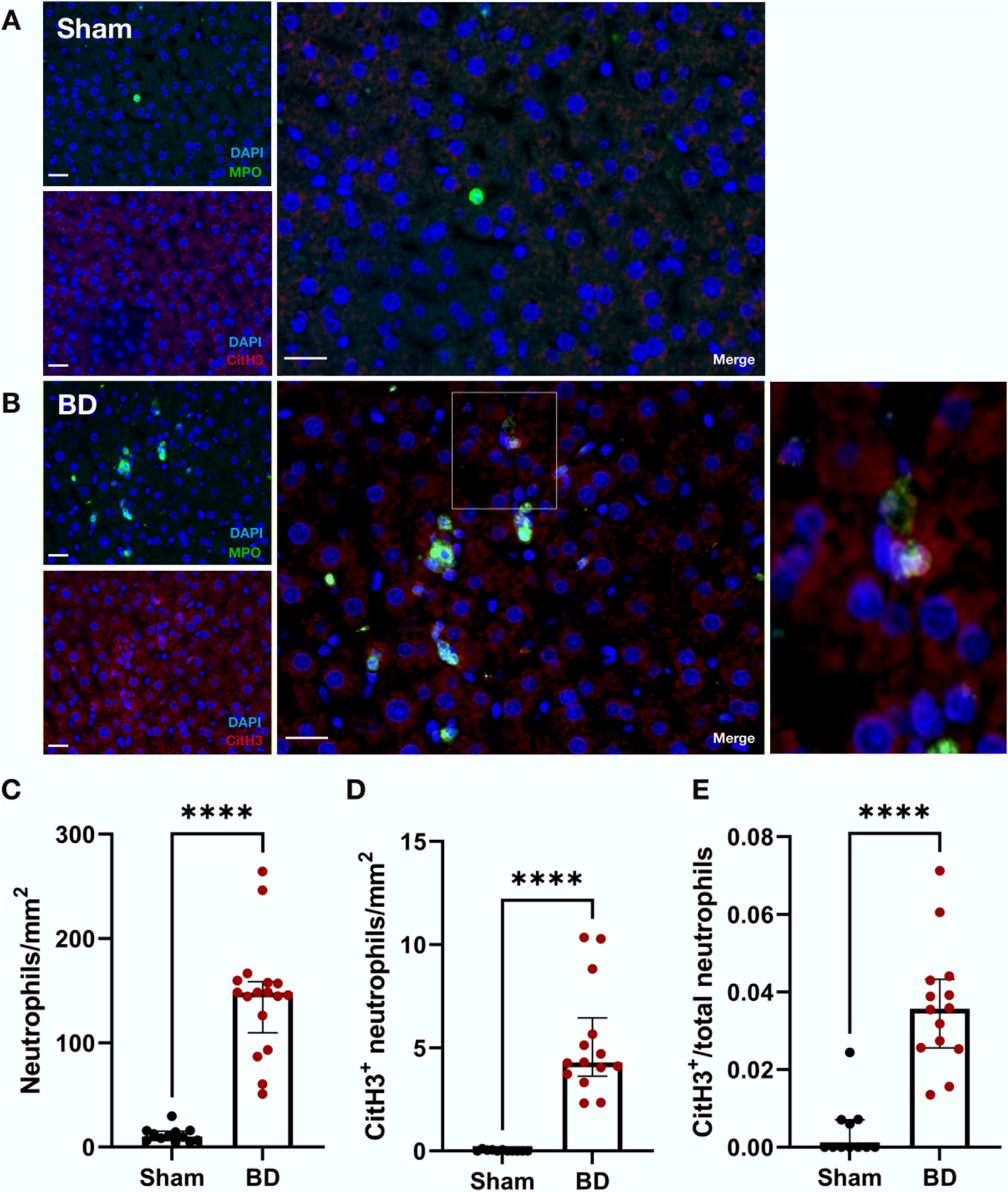
Neutrophil infiltration and NET formation in brain-dead rat livers. NET formation was observed in brain-dead rat livers **(B)**, while sham operated livers had little to almost no NET formation **(A)**. Brain-dead livers had significantly increased neutrophil infiltration **(C)**, CitH3 positive neutrophils **(D)** and CitH3 positive/total neutrophils ratios **(E)** compared to sham. Scale bar equals 20 µm. ****p < 0.0001. Data expressed as median (IQR). Abbreviations: BD, brain-death; CitH3, citrullinated histone 3; MPO, myeloperoxidase.

Livers in the BD group (Figure 7B) had significantly more neutrophils compared to sham (Figures 7A, C, p < 0.0001). The total number of CitH3-positive neutrophils (Figure 7D, p < 0.0001) as well as the NET/neutrophil ratios (Figure 7E, p < 0.0001) were both increased in livers from BD rats compared to sham-operated animals. In contrast to BD kidneys, only a small percentage of total neutrophils (3%-4%) were CitH3 positive in BD livers (Figure 7E). CitH3 positive neutrophils were mostly observed between hepatocytes in sinusoidal spaces (Figure 7B).

### Increased Endothelial Activation in Brain-Dead Rat Livers

**FIGURE 8:**
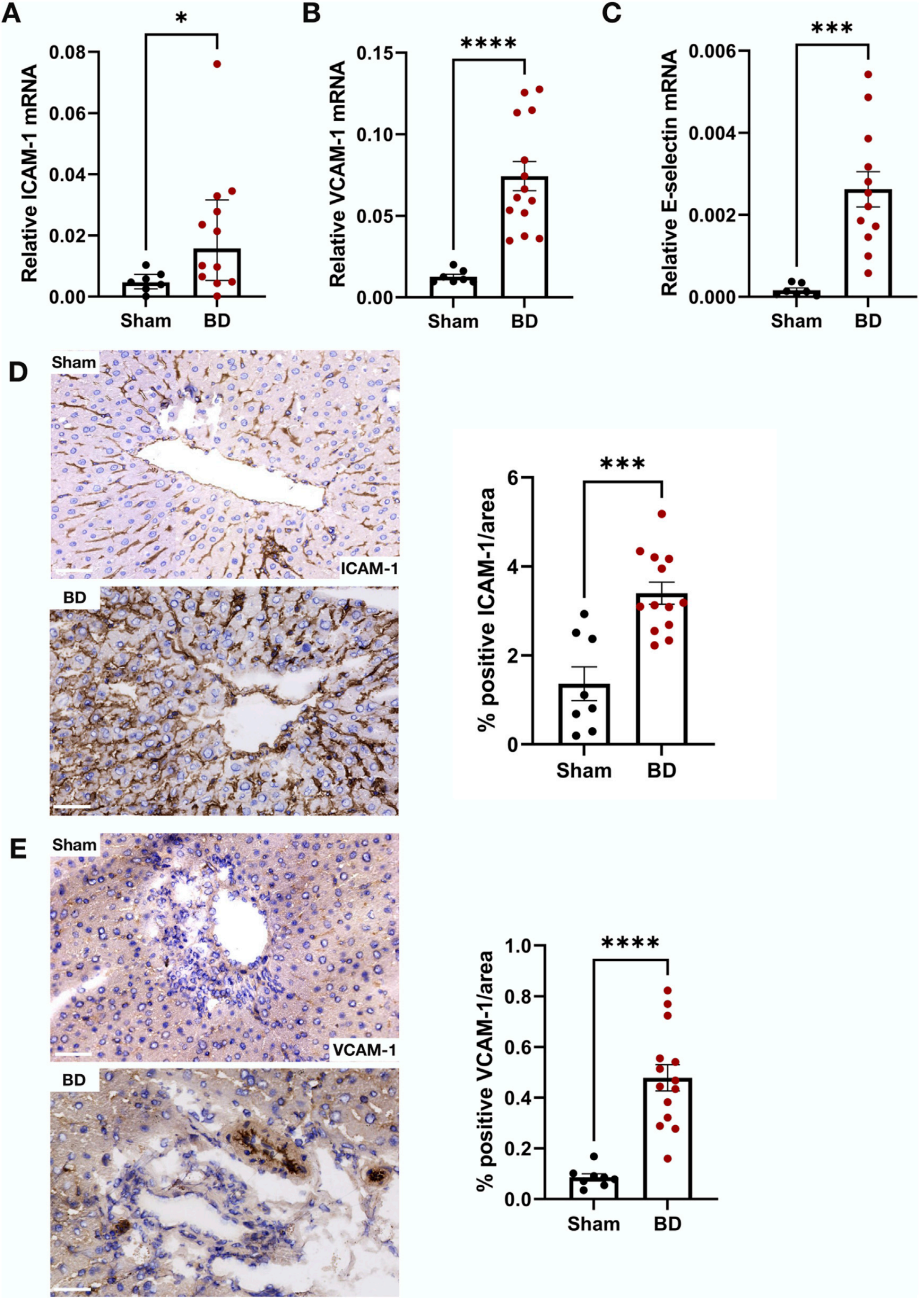
Endothelial activation in brain-dead rat livers. Brain-dead livers had increased ICAM-1 mRNA **(A)**, VCAM-1 mRNA **(B)** and E-selectin mRNA **(C)** expression and increased ICAM-1 **(D)** and VCAM-1 **(E)** protein levels. Scale bar equals 50 µm. *p < 0.05, ***p < 0.001 ****p < 0.0001. Data expressed as median (IQR), A; or mean (±SEM), B,C,D,E. Abbreviations: BD, brain-death; ICAM-1, intercellular cell adhesion molecule 1; VCAM-1, vascular cell adhesion molecule 1.

The mRNA levels of ICAM-1 (Figure 8A), VCAM-1 (Figure 8B) and E-selectin (Figure 8C) in BD rat livers were significantly higher compared to sham. BD livers also had increased ICAM-1 (Figure 8D) and VCAM-1 (Figure 8E) protein expression in the sinusoids and veins compared to sham. No significant correlation between endothelial activation markers and NET formation was observed in the livers (not shown).

### Brain-Dead Rat Kidneys and Livers Have Increased Macrophage Content

Since proinflammatory macrophages may be positive for MPO, the sections were also stained for macrophages (ED1) (Supplementary Figure S2) to distinguish NETs (NETs were CitH3^+^, MPO^+^, CD68^−^) from MPO-positive macrophages. BD kidneys (Supplementary Figure S2A) and livers (Supplementary Figure S2B) had increased macrophage content compared to sham-operated animals. Hearts, however, did not have significant infiltrated macrophages in the BD or sham-operated group (not shown). A comparison between kidneys and livers of BD rats (Supplementary Figure S2C) revealed significantly more CD68 (ED-1) positive macrophages in livers (p < 0.0001).

### Livers From Brain-Dead Rats Have Increased Platelet Infiltration

**FIGURE 9:**
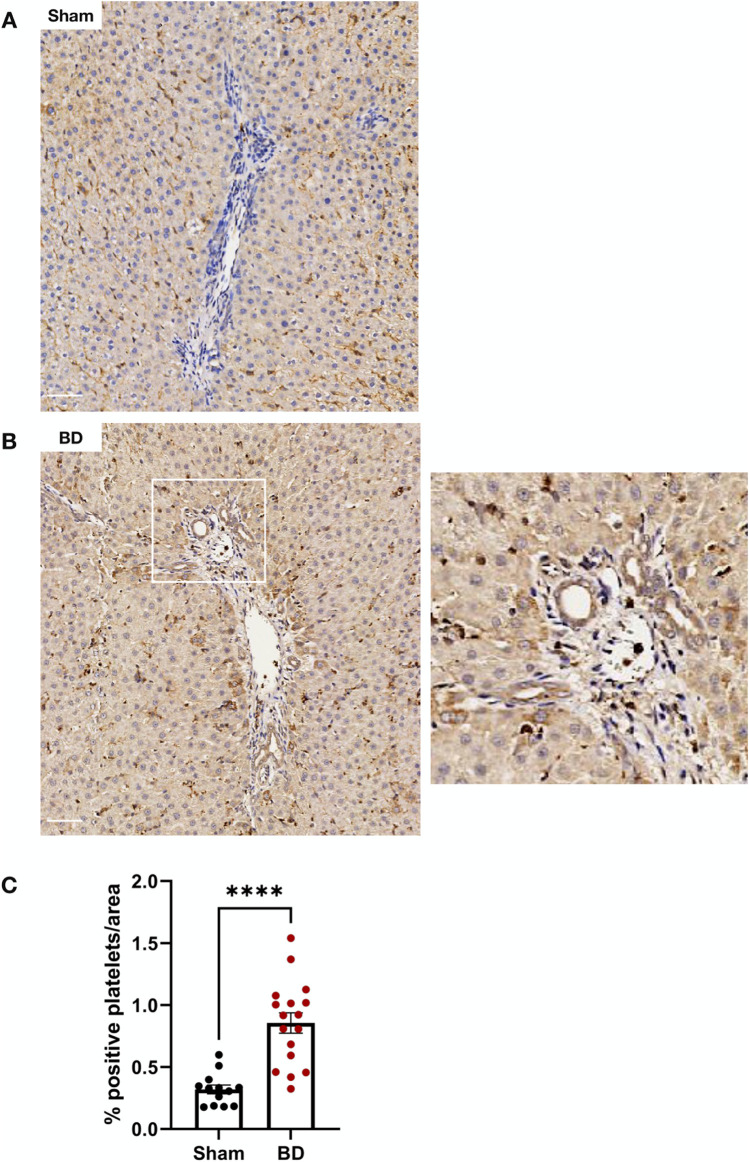
Platelet infiltration in brain-dead rat livers. Brain-dead rat livers **(B)** had increased platelet infiltration (CD41) compared to sham **(A)** quantified as percentage positivity per area analysed **(C)**. Scale bar equals 50 µm. ****p < 0.0001. Data expressed as mean (±SEM). Abbreviations: BD: Brain-death.

No platelet infiltration in kidneys and hearts from BD or sham-operated animals was observed (not shown). Liver sections from BD animals (Figure 9B) however, had increased numbers of platelets compared to sham-operated animals (Figures 9A, C).

### Male Brain-Dead Rat Hearts Have Increased NET Formation and Endothelial Activation

A comparison between males and females revealed no significant differences in NET formation (Supplementary Figures S3A–F) or endothelial activation (not shown) between sexes in BD kidneys (A-C) and livers (D-F). Sham male livers, however, had elevated neutrophils compared to females (Supplementary Figure S3D). BD male hearts had elevated numbers of neutrophils (Supplementary Figure S3G, p < 0.01), CitH3^+^ neutrophils (NET formation) (Supplementary Figure S3H, p < 0.0001), and ICAM-1 protein expression (p < 0.05) (not shown) compared to BD females, in contrast to the sham group which had no differences in NET formation (Supplementary Figures S3G–I) or endothelial activation (not shown) between sexes.

### Brain-Dead Rat Organs Have Differences in Cell Influx, NET Formation and Endothelial Activation

**FIGURE 10:**
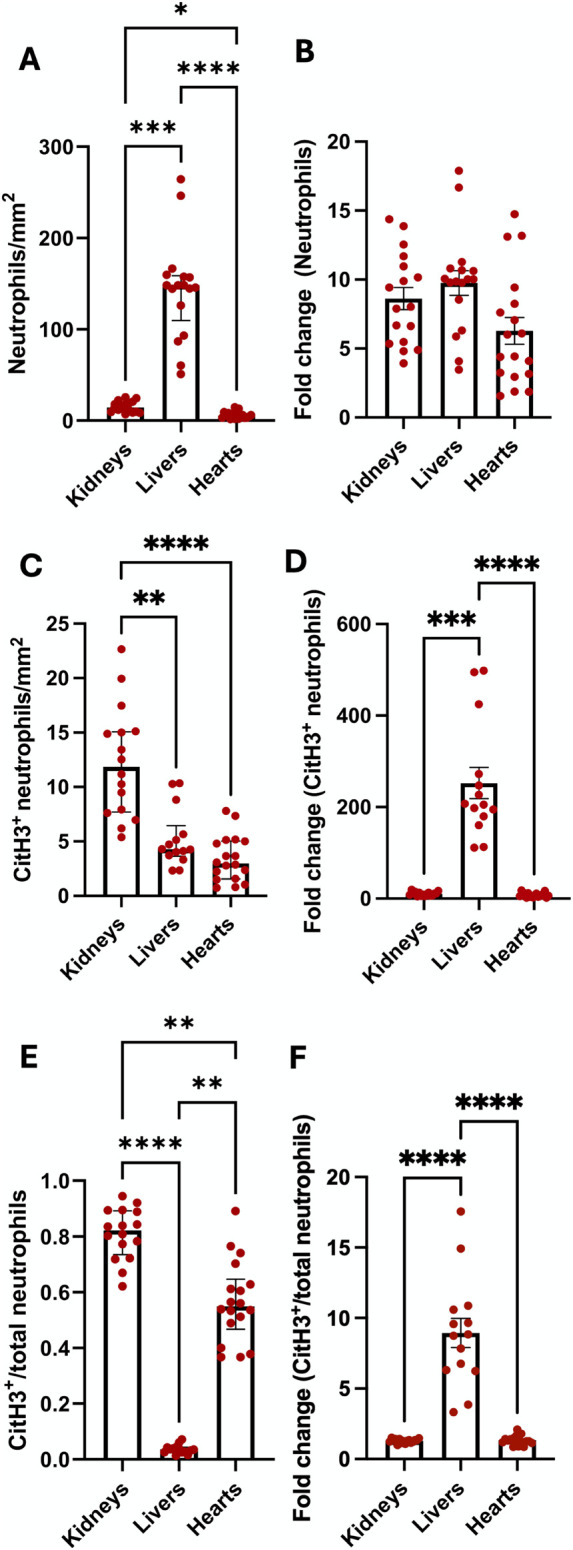
Differences in neutrophil infiltration and NET formation between organs. Brain-dead rat livers had the most infiltrating neutrophils, while kidneys had more neutrophils compared to hearts **(A)**. No differences in neutrophil fold change from sham operated animals were observed between the organs **(B)**. Compared to livers and hearts, kidneys had the highest level of NET formation **(C)**, however, livers had an increase in NET fold change, compared to kidneys **(D)**. NET/neutrophil ratios were higher in kidneys and hearts compared to livers, while kidneys had increased ratios compared to hearts **(E)**. Livers had increased fold change ratios compared to the other organs **(F)**. *p < 0.05, **p < 0.01, ***p < 0.001, ****p < 0.0001. Data expressed as median (IQR), **(A**, **C** and **E)** or mean ± SEM, **(B**, **D** and **F)**. Abbreviations: CitH3, citrullinated histone 3.

Compared to the kidneys and hearts, BD rat livers had significantly increased neutrophils, while BD rat kidneys had increased neutrophils compared to hearts (Figure 10A). However, when correcting for the numbers of neutrophils present in sham-operated rats (by expressing as fold-change in brain dead rats), no differences were observed between different organs (Figure 10B). BD kidneys had increased NET formation compared to both livers and hearts when expressed as absolute numbers of CitH3^+^ neutrophils (NETs, Figure 10C) as well as increased NET/neutrophil ratios (Figure 10E). Fold change of NETs and NET/neutrophil ratios was, however, higher in livers (Figures 10D, F). Distinct differences in endothelial activation in terms of mRNA and protein levels amongst kidneys, livers and hearts (Supplementary Figure S4) were also observed. BD rat kidneys and hearts had decreased ICAM-1 mRNA expression and fold change, compared to livers (Supplementary Figure S4A). BD hearts and livers had increased VCAM-1 mRNA compared to kidneys. No significant difference in VCAM-1 mRNA fold change was observed (Supplementary Figure S4B). Hearts had increased relative E-selectin mRNA expression, while both hearts and kidneys had increased fold change in E-selectin mRNA compared to livers (Supplementary Figure S4C). On the protein level, kidneys and livers had increased ICAM-1 compared to hearts, which was not reflected in fold change from sham (Supplementary Figure S4D). Kidneys also expressed increased VCAM-1 compared to livers and hearts, however, livers had increased fold change in VCAM-1 expression, compared to other organs (Supplementary Figure S4E).

### Brain-Dead Rats Have Increased Oxidative Stress

**FIGURE 11:**
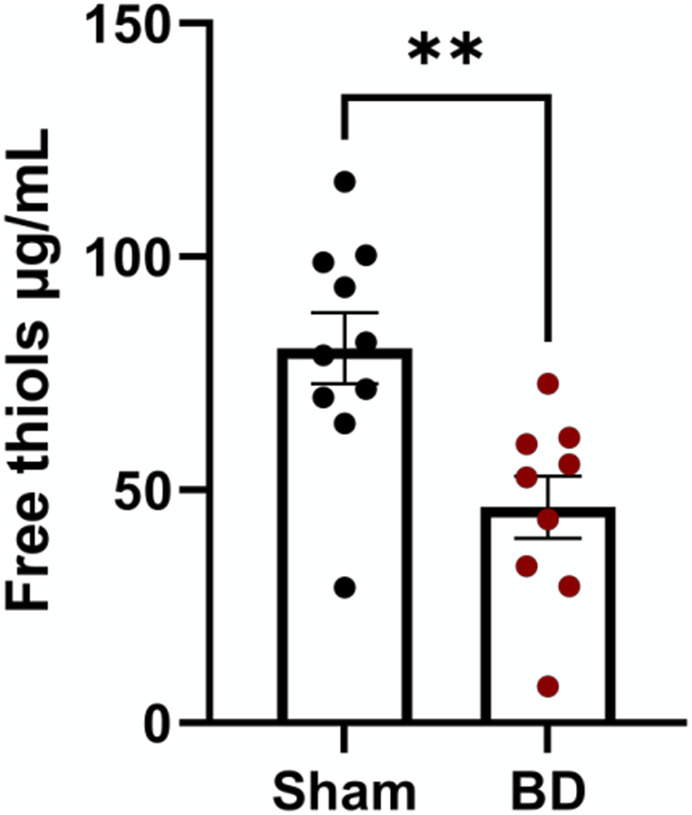
Reactive oxygen species in brain-dead rats. Brain-dead rats had increased circulating reactive oxygen species indicated by lower free thiol levels. **p < 0.01. Data expressed as mean (±SEM). Abbreviations: Brain-death.

Free thiol levels in plasma were lower in BD animals compared to sham-operated animals (Figure 11) reflecting increased oxidative stress in BD rats. Although free thiol levels in rat plasma tended to negatively correlate with NETs and endothelial activation in the organs analyzed (not shown), this association was not significant.

## Discussion

Neutrophils are the first responders of the innate immune system during inflammation and have a prominent role during different phases of transplantation [36]. This study demonstrated for the first time that NETs are already present in donor organs from a rat BD model. In this setting, the pro-inflammatory milieu during BD might prime neutrophils infiltrating the renal, cardiac, and hepatic tissues for NET formation. Given a previously established association between NET formation and organ injury [16, 21], this implicates a potential contribution of NETs towards compromised graft quality prior to reperfusion/transplantation. Moreover, associations with endothelial adhesion molecules and E-selectin might indicate a potential connection between NET formation and microvascular endothelial activation, particularly in the kidney, suggesting a role of NETs as contributing factor to organ microvascular endothelial dysfunction during brain-death.

The relation between NETs and organ injury has been explored by multiple groups, both through animal models [13, 16] and patient studies [27]. NETs are catalysts for heightened inflammation [13], coagulation, complement activation [24], the recruitment of additional immune cells and the augmentation of I/R injury [13]. Consequently, NETs have been implicated in lung [27], kidney [16], liver [13] and cardiac [37] injury in various diseases. NET components may enhance acute kidney injury (AKI) [16] and I/R-mediated liver injury [13]. In our study, the abundance of NET-forming neutrophils and accompanying increased endothelial activation suggest heightened injury, already in the donor, which is likely to worsen following reperfusion after transplantation.

Our model sheds light on differences between organs during brain-death. While the livers in brain death had increased neutrophil influx compared to kidneys and hearts, kidneys were found to have significantly increased absolute numbers of NETs and NET/neutrophil ratios compared to livers and hearts. Hearts also had increased NET/neutrophil ratios compared to livers. Increased NET and NET/neutrophil ratio fold change from sham was observed for livers compared to hearts and kidneys, but it should be considered that the liver had near zero NET formation in sham animals. Even though livers display the largest change in NET formation when comparing sham and BD, we believe the absolute number of NETs (as observed in kidneys) is most important in determining the potential effect of NET formation on graft outcome. Previous studies have shown differences in metabolic dynamics during brain-death between kidneys, hearts and livers [38, 39]. Kidneys and hearts were revealed to be more vulnerable to ischemic damage, compared to the liver, which is more resilient [38, 40]. Increased ischemic injury in kidneys and hearts might result in increased NET formation by the infiltrated neutrophils (reflected by NET/neutrophil ratios), making kidneys and hearts more vulnerable to cellular-mediated injury during brain-death. However, the impact of these differences between organs during brain-death needs to be further explored.

In the kidneys, the specific localization of cell infiltration and microvascular endothelial dysfunction (interstitium/peritubular capillaries and glomeruli) are often considered in the classification of rejection in the recipient. Recently, the profiling of innate immune cells in kidney grafts with rejection revealed an important role of innate immune cells in rejection, specifically in vasculature and glomeruli [41]. In our model, the abundance of NETs specifically located in the glomeruli might suggest a potential contribution towards rejection in the recipient by enhancing attraction of recipient immune cells to these compartments.

Endothelial activation in organs is an important indicator of graft quality as endothelial damage in deceased donors has been linked to early graft rejection [42]. During brain-death, the endothelium is activated through evolving inflammation and accordingly, inflammatory cytokine release and ROS production which leads to the expression of cell adhesion molecules and selectins on the endothelial cell surface [29, 43, 44]. In our model, an increase in the expression of kidney, heart and liver associated ICAM-1, VCAM-1 and E-selectin mRNA and protein, and increased ROS in the BD animals indicate a progression towards microvascular endothelial dysfunction during brain-death [7]. A positive correlation between ICAM-1, VCAM-1 and E-selectin, and NET formation suggest a potential relationship between increased NET formation and endothelial cell activation, especially in the kidneys, although a causal relationship needs to be explored in future experiments. An increase in the expression of cell adhesion molecules possibly contributed to increased neutrophil recruitment to organs in our model [45]. Infiltrated neutrophils, already primed for activation through inflammatory cytokines, complement, oxidative stress and coagulation parameters during brain-death might then be committed to NET formation through stimulation with DAMPs [16], inflammatory cytokines produced by other immune cells [17], existing NETs or activated endothelium [42]. NETs themselves also cause endothelial activation and damage [22, 31], therefore, during brain-death the NETs observed potentially also contributed to endothelial injury. The widespread detection of endothelial activation however likely suggests that brain death first inflicts endothelial activation/injury, before NET induced injury. It has previously been shown that endothelium from different organs are highly heterogeneous in terms of gene expression, protein and functional behavior [46]. This might shed light on the differences observed in endothelial activation between different organs and the relation with NETs during brain-death. Endothelial activation might occur earlier in the kidneys and hearts during brain-death, compared to the livers, particularly E-selectin expression which is associated with acute inflammation [47], or the kidney and heart endothelium might be more vulnerable to NET-related injury.

Given the established role of sex differences in the inflammatory response during BD [30, 48, 49] and previous published reports on differences in NET formation in other contexts [50], we also evaluated the effect of sex dimorphism in our model. In line with previous findings, BD kidneys and livers had no differences in neutrophil infiltration and NET formation between sexes [48]. However, in contrast to reports of increased leukocytes in female BD hearts and lungs [48], the male BD hearts in our study had significantly elevated neutrophils, NETs and ICAM-1 expression compared to female BD hearts. In previous studies, it has been shown that females have a greater inflammatory response compared to males during brain death [49]. This has been ascribed to a rapid fall in estradiol levels during BD, which is protective against heightened inflammation in healthy females [30]. A discrepancy between our results and previous findings could potentially be attributed to a difference in the BD model used. It has been demonstrated that fast induction of BD, used in previous reports [48], leads to greater hemodynamic instability and inflammation compared to slow induction of BD, used in our study, potentially implicating greater hormonal fluctuations, i.e., a more rapid fall in estradiol levels [51]. Interestingly, in other disease contexts such as multiple sclerosis, male patients had increased circulating NETs compared to females [50]. In contrast, *in-vitro* studies demonstrated increased lipopolysaccharide mediated NET release by neutrophils from females compared to males. 17β- estradiol had an inhibitory effect on NET formation in male derived neutrophils, but not in females [52]. These findings highlight the complexity of sex dimorphism on immune responses, suggesting that various other factors are also at play such as enzyme activity and sex differences in neutrophil biology. Whether sex dimorphism has a role in NET formation during BD, needs to be evaluated with follow up experiments. These findings however, do corroborate that sex might be an important consideration in the evaluation of donor hearts.

The lack of platelet deposition in organs, except for the livers, which had increased platelet deposition in the BD group, might suggest that the platelet-neutrophil interaction is not central to NET formation in our BD model or that platelet-neutrophil interactions occurred earlier during brain-death/microthrombi were dissolved through heparin administration [30].

A decrease in free thiol levels in the BD animals indicates increased oxidative stress in BD animals compared to sham-operated animals. Free thiols are a vital category of antioxidants found in plasma and protect cells and organs from oxidative stress by neutralizing ROS, therefore, a reduction in free thiol levels indicates an increased oxidative burden [53, 54]. Decreased free thiols in the BD group in this study are in line with previous work which identified a decrease in free thiol levels as a biomarker of oxidative stress following traumatic brain injury [54]. Decreased free thiol levels have also been associated with worse graft function in recipients following transplantation with deceased donor grafts [53]. Despite this, oxidative stress in the form of free thiol levels has not yet been characterized in the brain-dead donor. Our study is therefore the first to describe a drop in free thiol levels/oxidative stress during brain death. It has been established that both NET formation and endothelial dysfunction are associated with the production of ROS [33]. In the current study, although some animals with increased NETs also had decreased free thiols and therefore a higher oxidative burden (negative correlation) the association between free thiols and endothelial activation/NETs in the organs was not significant. This might suggest that other pro-inflammatory processes during the onset of brain-death (also) contributed to the formation of ROS [29].

A limitation of the study is that the relationship between endothelial activation and NET formation was indicated only through correlation and not through experiments in which mechanism could be established, i.e., *in-vivo* neutrophil depletion or *in-vitro* co-culture. To establish a causal relationship and mechanism, follow-up studies need to be performed, possibly including more *in-vitro* and *in-vivo* experiments to study the interaction between the endothelium, neutrophils and subsequent NET formation at different time points. Application of treatment in the BD animals against NET formation/neutrophil depletion can also shed light on the dynamics of endothelial activation and ROS formation and whether NETs are involved. The organs were also not transplanted. Future experiments should include a transplant group to evaluate the effect of NETs on functional parameters following transplantation.

In this proof-of-concept study, we have demonstrated for the first time that brain-death in a rat model induces NET formation and is associated with increased endothelial activation. NETs have already been shown to play a role in various stages of transplantation, but only hypothesized to be relevant already in the donor [12]. This study provides a basis for future research on clinical samples to establish whether the NETs might contribute to inferior graft quality observed to be associated with BD organs such as I/R injury, delayed graft function and early graft loss.

Currently, several therapeutics against NET formation already exist. In other disease contexts, PAD4 [55] or MPO inhibitors [56], various immunomodulatory drugs [57], and DNase-1 [58] have been proven successful to inhibit NET formation or resolve existing NETs. Testing these therapeutics either in the donor or during organ preservation should be considered as potential intervention strategies to attenuate organ injury.

## Data Availability

The raw data supporting the conclusions of this article will be made available by the authors, without undue reservation.

## Abbreviations

AKI, acute kidney injury; BD, brain-dead; CitH3, citrullinated histone 3; DAB, 3,3′-Diaminobenzidine; DAMPs, danger associated molecular patterns; FFPE, formalin fixed paraffin embedded; *g*, gravity; ICAM-1, intercellular adhesion molecule-1; IHC, immunohistochemistry; IF, immunofluorescence; I/R, ischemia/reperfusion; MPO, myeloperoxidase; NADPH, nicotinamide-adenine dinucleotide phosphate; NE, neutrophil elastase; NETs, neutrophil extracellular traps; PAD4, peptidyl arginine deiminase; ROS, reactive oxygen species; VCAM-1, vascular cell adhesion molecule.
