# Supplementary material for: Brain-Death in Rats Increases Neutrophil Extracellular Trap Formation in Donor Organs
Source: Transpl Int. 2025 Apr 22;38:14223. doi: 10.3389/ti.2025.14223 (PMC12052556; doi:10.3389/ti.2025.14223)
Supplement: Supplementary file 1 [file DataSheet1.DOCX]

**SUPPLEMENTAL DIGITAL CONTENT (SDC)**

**Details of the brain-death rat model**

Following anesthetization with 2 –5 % O_2_/isoflurane, brain-death was induced by inflating a balloon catheter between the dura and skull. Balloon inflation resulted in a hypotensive-, and subsequent hypertensive period, eventually leading to another hypotensive period when herniation into the brainstem occurs. The balloon inflation was stopped when the blood pressure became normotensive again (above 80 mmHg), representing the autonomic phase of brain death or sympathetic storm.[(1)](https://sciwheel.com/work/citation?ids=8098848&pre=&suf=&sa=0&dbf=0) During brain-death and maintenance, the rats were ventilated through tracheotomy and the blood pressure and temperature were maintained between 80-100 mmHg and 37°C, respectively. At the end of the brain-death period, rats were flushed with cold saline containing 250 U/ml heparin before procurement of the organs.

**Supplemental Table S1: Buffers and reagents used for immunofluorescence and immunohistochemistry**

| **Step** | **Reagent** | **Composition** | **Method** |
| --- | --- | --- | --- |
| Heat induced antigen retrieval | 0.1 M citrate buffer (pH 6.0) | 10.9 mM citric acid  Adjusted to pH 6.0 with 10N NaOH | 180 min at 60°C |
| Fixation of frozen sections | Acetone | Undiluted | 10 minutes at room temperature |
| Blocking endogenous hydrogen peroxidase activity | Hydrogen peroxide | 0.3 % hydrogen peroxide in PBS (*137 mM NaCl, 2.7 mM KCL, 10 mM Na_2_HPO_4_, 1.8 mM KH_2_PO_4_*) | 30 minutes at room temperature |
| Protein block | BSA | 5 % BSA in PBS (137 mM NaCl, 2.7 mM KCL, 10 mM Na_2_HPO_4_, 1.8 mM KH_2_PO_4_) | 1 hour at room temperature |
| Counterstain | Haematoxylin | Mayer’s solution: *3.3 mM Hematoxylin, 1 mM NaIO_3_, 210.8 mM*  *(NH_4_)Al(SO_4_)_2_, 252.7 mM C_2_H_3_Cl_3_O_2_ (50 g/L) and 5.2 mM citric acid.* | 1 minute at room temperature |
| Substrate for HRP | DAB | 1 g DAB and 0.03% hydrogen peroxide in 50 mL PBS | 10 min at room temperature |

Abbreviations: BSA, bovine serum albumin; DAB, 3.3'-Diaminobenzidine; HRP, horseradish peroxidase; PBS, phosphate buffered saline.

**Supplemental Table S2: Antibodies for immunofluorescence**

| **Antibody** | **Manufacturer** | **Stock concentration** | **Dilution in 1 % BSA/PBS** | **Method** |
| --- | --- | --- | --- | --- |
| **Primary antibodies** | | | | |
| Goat MPO (AF3667) | R&D Systems | 0.2 mg/mL | 1:200 | 90 minutes at room temperature |
| Rabbit CitH3 (ab5103) | Abcam | 0.6 mg/mL | 1:100 |  |
| Mouse CD68 (ED-1) | BIO-RAD | 0.1 mg/mL | 1:100 |  |
| **Secondary antibodies** | | | | |
| Donkey Anti-Goat IgG H&L (Alexa Fluor® 488) | Abcam | 2 mg/mL | 1:500 | 30 minutes at room temperature |
| Donkey Anti-Rabbit IgG H&L (Alexa Fluor® 647) |  |  |  |  |
| Donkey Anti-Mouse IgG H&L (Alexa Fluor® 568) |  |  |  |  |

Abbreviations: CitH3, citrullinated histone 3; MPO, myeloperoxidase

**Supplemental Table S3: Antibodies used for immunohistochemistry**

| **Antibody** | **Manufacturer** | **Stock concentration** | **Dilution in 1 % BSA/PBS** | **Method** |
| --- | --- | --- | --- | --- |
| **Primary antibodies** | | | | |
| Mouse anti rat ICAM-1 (1A29) | BD Pharmingen | 0.5 mg/mL | 1:100 | 1 hour at room temperature |
| Mouse anti rat VCAM-1 | Biogen | 3.6 mg/mL | 1:100 |  |
| Rabbit CD41(18308-AP) | Proteintech | 0.7 mg/mL | 1:500 |  |
| **Secondary antibodies** | | | | |
| Rabbit-anti-mouse-HRP polyclonal Ig  (P0260) | DAKO | 1.3 mg/mL | 1:100 | 30 minutes at room temperature |
| Goat-anti-rabbit-HRP polyclonal Ig (P0448) |  | 0.25 mg/mL |  |  |
| **Tertiary antibodies** | | | | |
| Goat-anti-rabbit-HRP polyclonal Ig | DAKO | 0.5 mg/mL | 1:100 | 30 minutes at room temperature |
| Rabbit-anti-goat-HRP polyclonal Ig  (P0449) |  | 0.5 mg/mL |  |  |

Abbreviations: ICAM-1, intercellular adhesion molecule; HRP, horseradish peroxidase; VCAM-1, vascular cell adhesion molecule 1.

**Supplemental Table S4: Specifications for qRT-PCR analyses**

| **Reagent** | **Manufacturer** | **Stock concentration** | **Method** |
| --- | --- | --- | --- |
| **RNA isolation** | | | |
| TRIzol™ | ThermoFisher Scientific | - | RNA isolation/ precipitation |
| Chloroform | Merck |  |  |
| Isopropanol | Merck |  |  |
| **cDNA synthesis** | | | |
| Random primers hexamers | ThermoFisher Scientific | 3 μg/mL | cDNA synthesis using random primers |
| 10 mM dNTP mix |  | 0.1 mM |  |
| 0.1 M DTT |  | 0.02 mM |  |
| RNaseOUT™ Recombinant Ribonuclease Inhibitor |  | 40 U/μL |  |
| SuperScript™ II Reverse Transcriptase |  | 20 U/μL |  |
| **qRT-PCR** | | | |
| Forward and reverse Primers | ThermoFisher Scientific | 3 μM | Light cycler PCR protocol with SYBR™ Green: 2 minutes 50°C, 10 minutes 95°C, 40 x cycles of 15 seconds 95°C and 60 seconds 60°C. |
| SYBR™ Green PCR Master Mix |  | 1x |  |

Abbreviations: cDNA, complementary DNA; dNTP, Deoxynucleotide Triphosphates; DTT, Dithiothreitol; qRT-PCR, quantitative real time polymerase chain reaction.

**Supplemental Table S5: Buffers and reagents for free thiol determination**

| **Reagent** | **Composition** | **Method** |
| --- | --- | --- |
| Plasma dilution buffer | 0.1M Tris (pH 8.2) | Diluted 1:4 |
| L-Cysteine standard | 10mM L-Cysteine in 0.1M Tris, 10 mM EDTA (pH 8.2) | Dilution from 15.625 to 1000 µM final concentration |
| DTNB | 1.9 mM DTNB in 0.1 M phosphate buffer (pH 7) | 20 minutes at room temperature |

Abbreviations: DTNB, 5.5′-dithiobis-(2-nitrobenzoic acid).


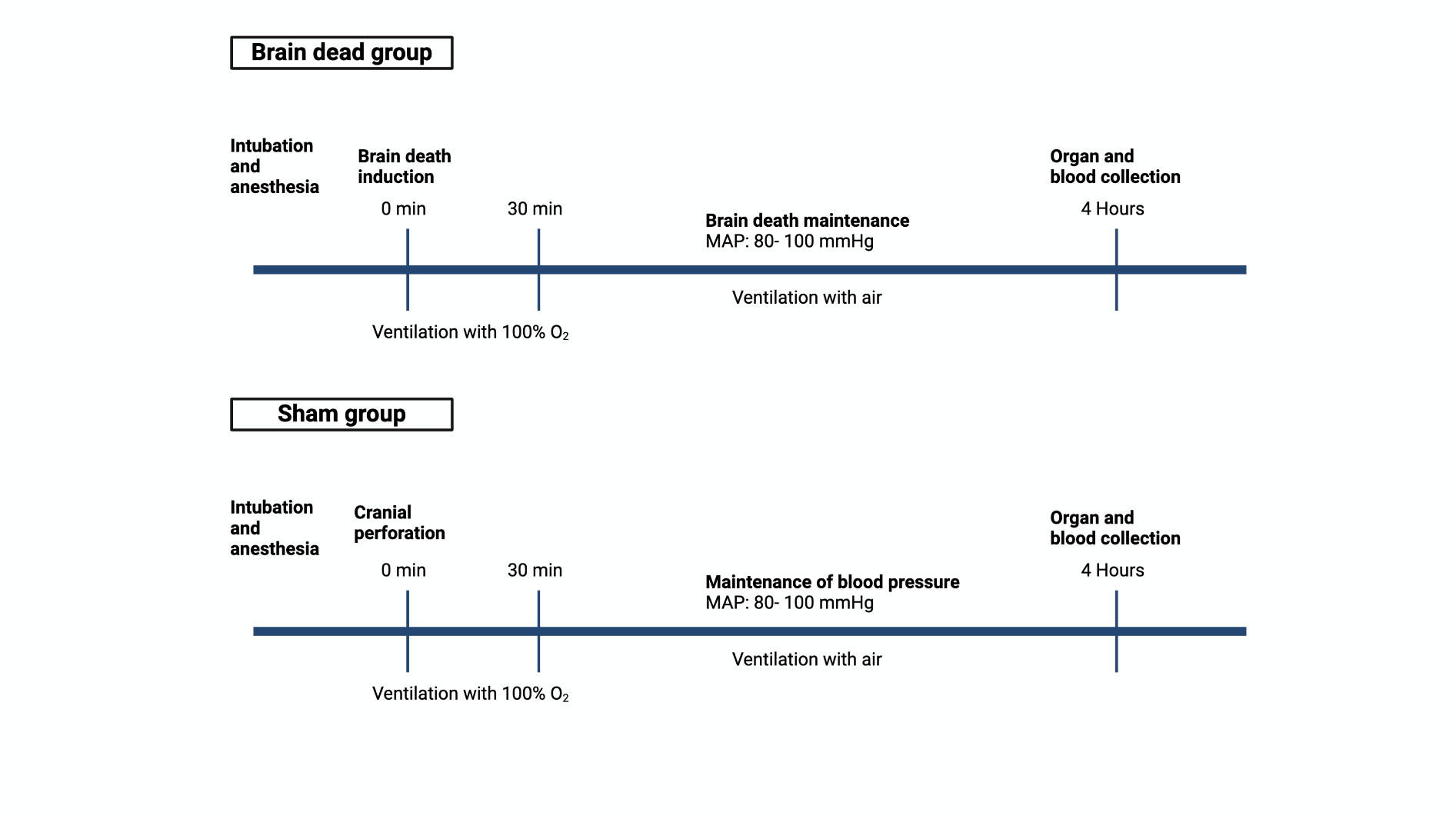
**Supplemental Figures**

**Figure S1: Brain-death rat model.** Brain-death was induced through inflation of an extradural placed balloon catheter. The rats were maintained for four hours before organs were flushed and procured. Following brain-death induction, the blood pressure was maintained at 80-100 mmHg. Sham operated animals received cranial perforation instead of brain-death and were maintained in the same way as the brain-dead animals. Tissue and plasma were processed for IHC, IF, qRT-PCR and free thiol level assays.

Abbreviations: IHC, immunohistochemistry; IF, immunofluorescence; MAP, mean arterial pressure; qRT-PCR, quantitative real time polymerase chain reaction.

**
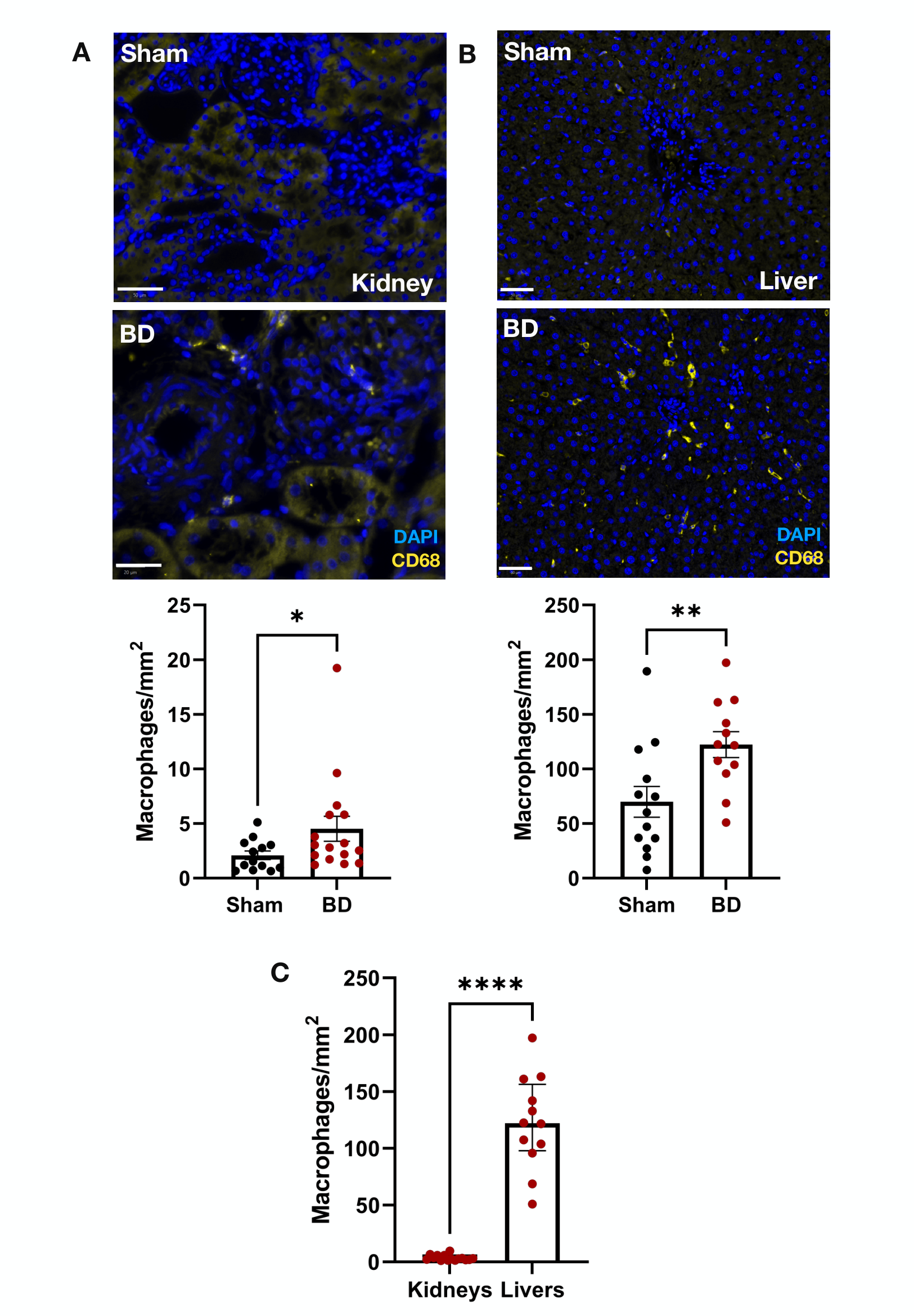
**

**Figure S2:** **Macrophage infiltration in brain-dead rat kidneys and livers.** Brain-dead rat kidneys (A) and livers (B) had increased macrophages (CD68) compared to sham operated animals. Brain-dead rat livers had increased macrophage infiltration compared to brain-dead rat kidneys (C). Scale bar equals 20 µm. *p< 0.05, **p<0.01, ****p<0.0001. Data expressed as mean ± SEM; A, B; or median (IQR); C.

Abbreviations: BD, brain-dead.


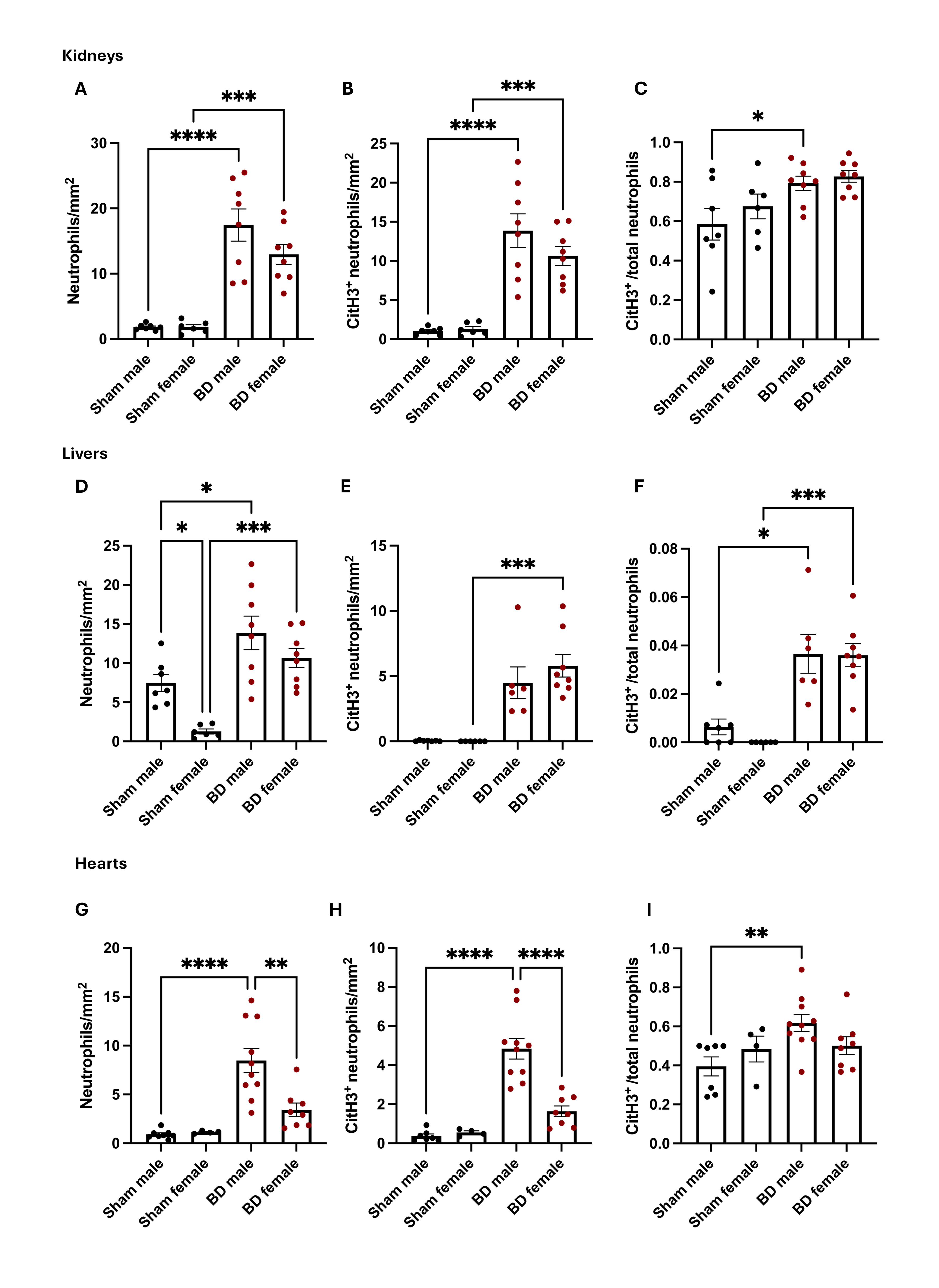
**Figure S3: Differences in NET formation between sexes.** Kidneys from male and female rats had no differences in neutrophil numbers (A), CitH3^+^ neutrophils (NET formation) (B), or NET neutrophil ratios (C). Sham male livers had increased neutrophils compared sham female livers (D). No differences between sexes was observed in brain-dead livers with regards to neutrophils (D), CitH3^+^ neutrophils (NETs, E) or NET/neutrophil ratios (F). Male brain-dead hearts had increased neutrophil infiltration compared to females (G) and increased NET formation (H). NET/ neutrophil ratios were not significantly different between sexes. *p<0.05, **p<0.01, ***p<0.001, ****p<0.0001. Data expressed mean ± SEM.

Abbreviations: BD, brain-dead; CitH3, citrullinated histone 3; NET, neutrophil extracellular traps.


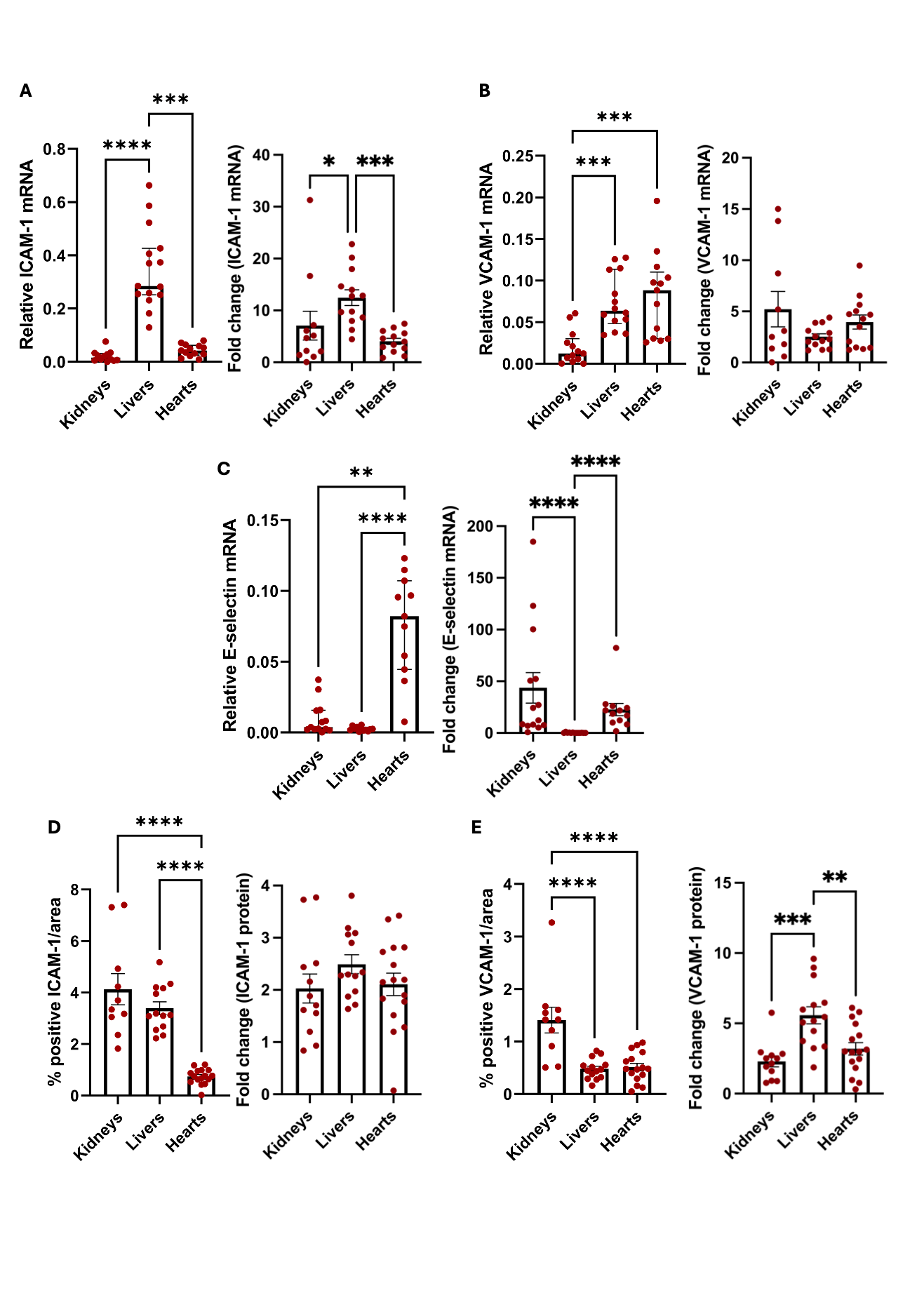
**Figure S4: Differences in endothelial activation between brain-dead rat organs.** Brain-dead livers had increased ICAM-1 mRNA expression and fold change compared to kidneys and hearts (A). Brain-dead rat livers and hearts had increased VCAM-1 mRNA expression compared to kidneys (B). Brain-dead rat hearts had the most absolute E-selectin mRNA expression, but both kidneys and hearts had increased fold change of E-selectin compared to livers.(C). Brain-dead rat kidneys had the highest ICAM-1 (D) and VCAM-1 (E) protein levels. Brain-dead livers, however, had increased ICAM-1 protein compared to hearts and higher fold change in VCAM-1 protein compared to kidneys and hearts. *p<0.05, **p<0.01, ***p<0.001, ****p<0.0001. Data expressed as median (IQR); B, C; or mean (SEM); A, D, E. Abbreviations: ICAM-1, intercellular adhesion molecule 1; VCAM-1, vascular cell adhesion molecule 1.

**References**

[1.    Kolkert JLP. ’t Hart NA. van Dijk A. Ottens PJ. Ploeg RJ. Leuvenink HGD. The gradual onset brain death model: a relevant model to study organ donation and its consequences on the outcome after transplantation. Lab Anim. 2007 Jul;41(3):363–71.](https://sciwheel.com/work/bibliography/8098848)
